# Designing an education intervention for understanding racism in healthcare in Sweden: development and implementation of anti-racist strategies through shared knowledge production and evaluation

**DOI:** 10.1177/14034948211040963

**Published:** 2021-09-11

**Authors:** Hannah Bradby, Sarah Hamed, Suruchi Thapar-Björkert, Beth Maina Ahlberg

**Affiliations:** 1 Department of Sociology (incl. Centre for Social Work (CESAR)), Uppsala University, Uppsala, Sweden; 2 Department of Government, Uppsala University, Uppsala, Sweden; 3 Skaraborg Institut, Skövde, Sweden

**Keywords:** Healthcare practitioners, racism, participatory methods

## Abstract

An educational intervention, based on qualitative evidence of racism in healthcare, is described. Using vignettes from a previous project, interviews were conducted to gather qualitative evidence of racism in healthcare settings from a wide range of healthcare staff in Sweden. From this interview material, case studies were devised that were subsequently presented to trainee healthcare professionals, in a seminar discussion. After the seminar, trainees responded to reflective questions. The order of work, as well as the materials used, are described. This intervention was successful in facilitating discussion about racism in an educational context, despite the difficult nature of these conversations for some participants.

## Rationale for the study

This article describes an educational intervention in healthcare training, discussing racism in healthcare as part of a larger Swedish Research Council funded project. Sweden’s image as an egalitarian, tolerant country that welcomes refugees and offers moral leadership on a global stage has hindered a national discussion about racism in public services. A longstanding policy of colour-blind welfare universalism transcends the particularity of the needs, experiences and perspectives of specific population groups in Sweden [1], that may be designated in terms of their minority culture, ethnicity or migrant background, and is assumed to negate the need for anti-racist strategies. Evidence of the harms of racism in healthcare settings for both patients and staff [2–4] underlines the need to investigate how to undo racism in healthcare settings [5]. Overt racism of the type that was scientifically defended prior to the Second World War [6] is not only illegal but also socially unacceptable in most public healthcare settings, whereas more subtle, invisible structural forms of racialised discrimination, which do not involve the identification of individual racists [7], are more widespread. Racism in healthcare settings, whether it is overt or invisible, contravenes the principles of an equitable public health system, by damaging the wellbeing of patients and of staff and by disrupting equitable access to good quality care. As there is little systematic data gathering around racialised groups or the experience of racism, the extent of this racism cannot be stated. Sweden’s excellent register data notwithstanding: migration background and foreign birth are collected, but no data based on ethnic or racialised categorisation exist. As a first step towards being able to address racism in healthcare settings, we describe how we designed an educational intervention based on interview material about healthcare professionals’ experience of racism in healthcare. Our aim was not to devise a unified terminology or specify recommended definitions, but rather, in recognition of the personal and political difficulties of naming and discussing racism in healthcare, to find a way of speaking constructively about its effects. The positive outcome of this process was the creation of a space to discuss racism between healthcare professionals without fear or reproach.

## Design

Here we describe how we designed an educational intervention, which is addressed in more detail elsewhere [5]. As this work was participatory and qualitative, exploring whether and how racism can be discussed in a range of different healthcare settings, the design was emergent, even though it was planned prospectively. This meant that each stage emerged from the outcome of the previous stage, taking into account what research participants said and how they said it.

Between June 2017 and February 2020, we interviewed a total of 58 healthcare professionals from a range of professional and geographical settings. Initially we had planned to convene a set of focus groups and this was possible in some cases. Group interviews were convened with colleagues who had worked together and knew one another already. When professionals were unwilling or unable, due to other demands, to speak in front of their colleagues, we conducted one-to-one or paired interviews. In paired and group discussions, participants’ meanings emerged through their interactions with each other, as well as with the interviewer, which gives insight into which ideas are shared and agreed on, as well as where opinions differ between individuals.

Oral and then written consent for participation was obtained, after careful delineation of the aims and objectives of the research study. During the interviews the questions shown in Supplemental Appendix 1 were posed and, as is usual in semistructured interviews, the research participant was able to steer the conversation to some extent. The word racism was used in the interview questions, but the interviewer did not offer a definition of the term. The research participants used the terminology that they felt was most suitable, and the interviewer sought to clarify their meaning through further questions. As the topic of racism is sensitive and, in some cases, healthcare professionals found it difficult to share their thoughts, we used some of the narratives on racism shared with us in an earlier project [3] to construct vignettes. Vignettes have been used in health research in combination with interviews to explore difficult and sensitive public health issues such as HIV testing [8] and mental health issues [9] but also in research exploring racism [10]. The vignettes were used in our research as icebreakers and as a tool to guide the discussion and included stark instances of racial practices as well as more ambiguous moments in which clinical or resource allocation priorities seemed to be incompatible with egalitarian anti-racist practice.

The interviews were audio-recorded and transcribed into text files. This material was imported into Atlas-ti and coded. The coding scheme was devised, tested and revised twice by being applied to interview transcripts by two authors independently.

Using this coding scheme, all the accounts of racism or incidents that might appear as racist in nature were identified in the interview material and the material was re-written as three case studies. This involved writing a summary of the incident that was described in the interview, so as to make it comprehensible to readers and to ensure that no identities were revealed. In collaboration with two healthcare service training programmes, two of these case studies were presented as part of university-level teaching. Students were given a lecture on racism in Sweden generally and in healthcare in particular, and a short article to read by way of preparation for participation in a seminar discussion. The lecture and the article described ways that racism might manifest, but did not emphasise a singular definition. The details of the case studies were given to the students step-wise during the seminar, as shown in Supplemental Appendix 3, so as to allow discussion to unfold slowly and to encourage students to share their own experiences.

After the guided discussion, students were asked a number of reflective questions – see Supplemental Appendix 4 – to which they provided written responses, together with signed consent forms. An evaluation of the seminar is reported on elsewhere (Odzakovic et al. under review).

## Population and sample size considerations

In gathering material for qualitative analysis, the aim is to get enough material to reflect both the range of relevant experience and to be able to see the common ground shared across individual cases. There is no specific requirement for numbers of interviews to be collected, individuals or cases to be contacted. However, qualitative researchers often talk about data saturation as the point where familiar stories recur, new interviews or observations are being made [11].

Important in this approach is that the case studies we designed for the educational intervention represent a wide range of healthcare staff, including one or more nurse, auxiliary nurse, doctor, dentist, medical student, midwife, dental hygienist, psychologist and public health specialist. The interviews were sampled from big city and small town settings, with a range of ages, ethnicities and genders represented. Crucially for a discussion of racism, while all the participants were Swedish citizens, they had a range of backgrounds in terms of including both native Swedes and people of migrant background. In our case, 58 people employed in healthcare described their experience of racism in a professional setting. This offers the possibility of designing different case studies or vignettes for future educational interventions in different medical settings.

Another consideration in the sample size was the reluctance that we encountered to speak about racism at all. During the first 18 months of the project, we struggled to persuade individuals and organisations to cooperate with our project. This reluctance shifted during the early months of 2020, when the COVID-19 pandemic prevented travel, yet we nonetheless experienced great interest in our study, perhaps as a result of the transnational Black Lives Matter (BLM) protests mobilising ideas on structural racism, together with future visions of racial and ethnic equality. We began to receive numerous invitations, from different organisations and higher education institutions to communicate our research, which made it easier to validate our findings. That is, in presenting our work to, for instance, healthcare professional and student associations, we got direct feedback as to people’s recognition of the stories of racism and confirmation that they too had had similar experiences.

Once the case studies were refined, cooperation with a healthcare training programme allowed structured discussions with trainee clinicians to be convened. Due to the ongoing pandemic, the educational interventions were convened via zoom. Although it is hard to know whether discussions around racism would have been easier to conduct in person or not, it is our experience that students find it harder to discuss seminar topics on sensitive and politicised issues virtually. This project is in the process of negotiating future training programmes, both nationally and internationally.

## Basic characteristics of the study

By using conversation-based methods, this study has succeeded in interviewing a diverse sample of healthcare staff about racism and in using this to create an evidence-based material for use in collaborative and reflective conversations about racism convened with healthcare trainees.

The interviews were informative despite some initial reluctance to discuss racism.

The students who have participated in structured discussions based on case studies were able to discuss racism in healthcare, even though it had not been a topic of their previous training or work-based professional training. Supporting students’ ability to discuss racism with their colleagues should allow the development of better workplace communication, contributing to reflective professional practice, improved competency in treating patients and the identification and tackling of organisational routines that have a racist effect.

The reflections that students have submitted from two educational interventions in two medical faculties are the basis of an ongoing qualitative content analysis to understand the effect of the intervention. These will be published separately from this protocol description. It is anticipated that our ongoing evaluation of the intervention with student nurses may be applied to other health and social care professionals in due course.

This success in discussing racism openly and constructively among Swedish healthcare professionals and trainees is worth noting because it has so rarely been undertaken before in a structured fashion.

## Supplemental Material

sj-docx-1-sjp-10.1177_14034948211040963 – Supplemental material for Designing an education intervention for understanding racism in healthcare in Sweden: development and implementation of anti-racist strategies through shared knowledge production and evaluation Click here for additional data file. Supplemental material, sj-docx-1-sjp-10.1177_14034948211040963 for Designing an education intervention for understanding racism in healthcare in Sweden: development and implementation of anti-racist strategies through shared knowledge production and evaluation by Hannah Bradby, Sarah Hamed, Suruchi Thapar-Björkert and Beth Maina Ahlberg in Scandinavian Journal of Public Health
