# Supplementary material for: Designing an education intervention for understanding racism in healthcare in Sweden: development and implementation of anti-racist strategies through shared knowledge production and evaluation
Source: Scand J Public Health. 2021 Sep 11;51(4):531–4. doi: 10.1177/14034948211040963 (PMC10259079; doi:10.1177/14034948211040963)
Supplement: sj-docx-1-sjp-10.1177_14034948211040963 – Supplemental material for Designing an education intervention for understanding racism in healthcare in Sweden: development and implementation of anti-racist strategies through shared knowledge production and evaluation [file sj-docx-1-sjp-10.1177_14034948211040963.docx]

Appendices (three in total, that could be included as Supplementary material)

Appendix 1

Interview schedule for healthcare providers

1. Can you describe a typical work day?
   1. What challenges do you face during your work?
   2. Did the dynamic in your work place change after the arrival of migrants in 2015? If so, how?
   3. Have you had a complaint of racism made against you?
      1. If so, can you tell us what happened? Describe the incident – the context, participants, consequences etc. Did anything change after the complaint?
   4. Have you felt that a lack of trust from a patient on the grounds of your ethnicity?
      1. Describe what happened and how you responded.
   5. Have you felt seriously disrespected* by colleagues in healthcare for instance due to cultural differences? Describe what happened. Did you change anything after this incident?

*translation of the Swedish 'kränkt’

Appendix 2

Case studies and student questions presented to trainee healthcare providers as an educational intervention

**Case study 1**

Part 1

Eva, a nurse who works on the children’s ward at a Swedish Hospital, feels stressed, as usual. There are many patients and a long waiting list. The parent of a child who was due to have a MRT scan rings to cancel the appointment.

“That doesn’t often happen!” thinks Eva, but it’s good to have space so we can book in a child from the waiting list. Eva finds the waiting list and starts going through it.

“OK, who needs to get this appointment,” says Eva to herself, as she wonders how to prioritize.

“Hmm, I should take the most urgent case first” thinks Eva.

Then Eva goes through the list and sees that there are many patients with foreign names and many need a translator as the parents cannot speak Swedish well.

Eva sighs. “Translators are such hard work” she thinks.

**Student questions:**

- What are the grounds for prioritizing patients for healthcare?
  - - Should one prioritize only from medical considerations? If not, what other priorities should play a role?
    - What are one’s responsibilities to use resources wisely?

Part 2

“Translators are such hard work” sighs Eva. “The whole business of booking a translator and then it has to be the right translator. Sometimes it’s impossible to get hold of the translator…”

After pondering for a while, Eva decides to leave aside the patients that need a translator.

“Hmm, that doesn’t feel quite right,” thinks Eva to herself.

“But I have also looked at the urgency of the cases” reasons Eva.

“So, I haven’t done anything wrong … I have no time to sit here and book the translator as well.”

In the end Eva rings a patient with a Swedish background and books an appointment.

**Student questions:**

- Did Eva do the right thing? If not, why? What do you think?
  - - Is it discriminatory to not prioritise a patient who needs a translator?
    - Is it racism to not prioritise a patient who needs a translator?

**Case 2**

Part 1

A Swedish Doctor, Omar, sees that he has a patient who might have hemorrhoids and that may require an operation. Today patients are coming for an initial investigation to decide whether or not to operate. Omar is used to doing hemorrhoid operations.

Omar goes to reception and calls the patient.

“Tobias Lindegren!” calls Omar and waits for an answer.

No-one answers.

“Strange, I saw that the patient had registered himself.” Omar thinks to himself “Perhaps he’s at the toilet.”

Tobias Lindegren is sitting in the waiting room and sees Omar arrive and call for him. He doesn’t answer Omar and after Omar has left the waiting room, Tobias goes to the nurse, Stina who is sitting in reception and says “I don’t want to be treated by a black-skull*. Have they taken over Sweden now? I want a Swedish doctor who can pronounce my name properly.”

*****svartskalle in Swedish

**Student questions:**

- What is meant by a ‘Swedish doctor’? What word would you usually use to describe a person who is white?
- What would you have done in Stina’s place?
- Have you seen anything similar? If so, what happened?

Part 2

The nurse, Stina, sighs. She sometimes has patients who want ‘A Swedish doctor’. Then she usually books those patients with a Swedish doctor. Stina cannot bear the fuss!

Tobias is given a time with ‘A Swedish doctor’, Jonas. Stina writes up what has happened in the patient’s notes. She tells Omar what has happened. It is not the first time that Omar has had this experience.

“It’s not possible to talk about this kind of thing … best just to keep working” thinks Omar to himself.

Jonas meets the patient Tobias. After an investigation, Jonas decides that Tobias needs an operation. He tries to persuade Tobias that Omar is a good doctor and the best person to undertake the hemorrhoid operation.

After a long discussion, Tobias says:

“OK then, he can treat me, but you must be there as well.”

“OK,” agrees Jonas.

Omar accepts this. In the end both Omar and Jonas perform Tobias’ operation. After this incident, Tobias continues with Omar as his doctor. They become friends.

**Student questions:**

- How has this case been handled?
  - What is Tobias’ responsibility?
  - What is Stina’s responsibilty?
  - What is Omar’s responsibility?
- How can we address racism in healthcare?
  - Is your opinion influenced by the fact that Omar and Tobias became friends?
  - Is it possible to be friends with patients that express themselves as racists?

Appendix 3

Reflection questions for students after experiencing the educational intervention

1. Have you ever discussed racism in care at your workplace with your colleagues and / or with your patients? If not, why do you think that racism is not discussed? If yes, can you briefly describe how these discussions took place?
2. What do you think is the most interesting or surprising about the seminar discussion today?
3. On a scale from 0 – 5, how comfortable is it for you to speak about racism in care? (0 = I would not want to talk about racism in care and 5 = I feel quite comfortable speaking about racism in care). Justify your response!
4. Do you feel that this seminar has contributed to a better understanding of racism in care?
